# Hypoxia activates the unfolded protein response signaling network: An adaptive mechanism for endometriosis

**DOI:** 10.3389/fendo.2022.945578

**Published:** 2022-10-20

**Authors:** Yong Zhou, Yang Jin, Yuan Wang, Ruijin Wu

**Affiliations:** Women’s Hospital, Zhejiang University School of Medicine, Hangzhou, China

**Keywords:** hypoxia, unfolded protein response, endometriosis, endoplasmic reticulum stress, hypoxia-inducible factor

## Abstract

Endometriosis (EMS) is a chronic gynecological disease that affects women of childbearing age. However, the exact cause remains unclear. The uterus is a highly vascularized organ that continuously exposes endometrial cells to high oxygen concentrations. According to the “planting theory” of EMS pathogenesis, when endometrial cells fall from the uterine cavity and retrograde to the peritoneal cavity, they will face severe hypoxic stress. Hypoxic stress remains a key issue even if successfully implanted into the ovaries or peritoneum. In recent years, increasing evidence has confirmed that hypoxia is closely related to the occurrence and development of EMS. Hypoxia-inducible factor-1α (HIF-1α) can play an essential role in the pathological process of EMS by regulating carbohydrate metabolism, angiogenesis, and energy conversion of ectopic endometrial cells. However, HIF-1α alone is insufficient to achieve the complete program of adaptive changes required for cell survival under hypoxic stress, while the unfolded protein response (UPR) responding to endoplasmic reticulum stress plays an essential supplementary role in promoting cell survival. The formation of a complex signal regulation network by hypoxia-driven UPR may be the cytoprotective adaptation mechanism of ectopic endometrial cells in unfavorable microenvironments.

## Introduction

Endometriosis is a common benign gynecological disorder characterized by the presence and growth of functional endometrial glands and stroma outside the uterine cavity, usually accompanied by reactive fibrosis and muscular metaplasia of affected organs (1, 2). Unfortunately, only a few studies estimated the prevalence and incidence of endometriosis in the general population, reporting that the prevalence of symptomatic endometriosis is about 10%, and approximately 2-7/1000 women are diagnosed with endometriosis every year. However, 11% of cases remain undiagnosed (3, 4).

EMS is closely related to dysmenorrhea, painful intercourse, painful defecation, and infertility in women of reproductive age and may seriously affect their quality of life (5, 6). In addition, patients and physicians face the great challenge of high recurrence rates after conservative EMS surgery (7, 8). Lesions are most commonly found in the pelvic cavity, including the ovaries, uterus, sacral ligaments, vaginal septum, bladder, rectum, and ureters. EMS may also invade organs other than the pelvic cavity, such as abdominal incision (9), diaphragm, and even lungs (10).

## Theoretical basis of hypoxia in EMS

The exact pathogenesis of EMS is still unclear. However, the implant theory is still widely accepted as the mainstream theory (11). The hypothesis is based on the concept that EMS lesions’ matrix and glands are derived from ectopic endometrium. Thus, EMS is considered a benign metastasis of ectopic endometrium, which is transferred from the uterine cavity to another position in the body through different pathways. The theory of menstrual blood reflux, based on clinical and anatomical observations, was first proposed by Sampson in 1927 (12). It is believed that most EMS were induced by endometrial fragments entering the pelvic cavity through fallopian tube reflux during menstruation, the transition from the endometrium to ectopic endometrium, and growth on peritoneum as well as ovary.

Although menstrual reflux theory cannot explain all forms of EMS, it remains the most accepted hypothesis of EMS. Some scientists found that 76~90% of women have the phenomenon of menstrual blood regurgitation of fallopian tubes (13), and laparoscopy has revealed that endometrial epithelial cells can be isolated from abdominal fluid at the early stage of endometrial hyperplasia (14). In addition, some researchers have identified viable single endometrial cells and glandular structures from the shedding of menstrual blood (15). The “eutopic endometrium determinism”, proposed by Lang (16), believes that the onset of EMS depends on the characteristics of eutopic endometrial cells in the uterine, so eutopic endometrial cells of patients with EMS may have stronger survival and proliferation ability after falling off. Thus, they are more likely to become ectopic lesions. Among this process, retrograde menstruation is the only bridge from the potential pathogenicity of endometrial cells to the onset of EMS. Recently, based on the physiological phenomenon of periodic stripping and regeneration of endometrium, it is speculated that endometrial stem cells with differentiation potential may exist in menstrual blood. Therefore, some scientists proposed the “stem cell theory” of EMS pathogenesis (17). Subsequently, relevant studies also confirmed that after menstrual blood enters the pelvic cavity from the fallopian tube, endometrial cell fragments with stem cell characteristics can adhere to mesothelial cells and promote the growth of EMS lesions, leading to the occurrence and development of EMS (18, 19). These theories enriched the theoretical basis of planting theory.

**Figure 1:**
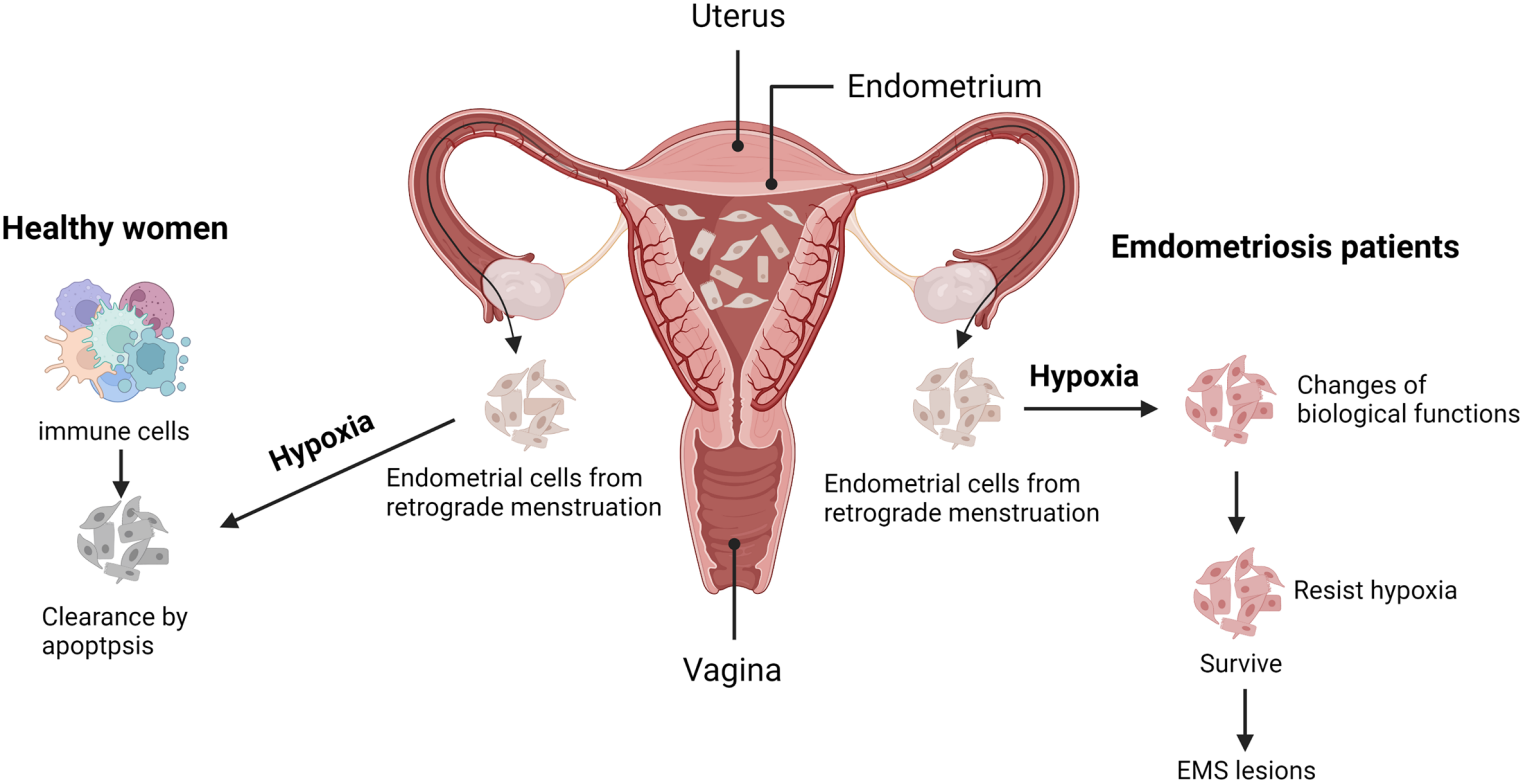
Possible adaptive mechanism for endometriosis under hypoxia. During menstruation, the exfoliated endometrium fragments flow into the pelvic cavity with retrograde menstruation. Under normal circumstances (as shown in the left part), the shedding endometrial cells will face acute hypoxic stress, activating apoptotic signals and being cleared by the immune cells. Conversely, those endometrial cells of endometriosis (EMS) patients (as shown in the right part), possess with altered biological functions, may resist hypoxia or apoptosis and finally develop into EMS lesions. Created with BioRender.com.

The anatomical characteristics of the uterine vessels are that the body branches of the uterine arteries vertically discharge the arch artery along with the muscle layer of the lateral wall of the uterus. The uterine spiral artery then extends vertically into the endometrium, finally forming a blood-rich capillary network in the endometrium (as shown in **Figure 1**). During menstruation, endometrial vasospasm causes acute hypoxia of the endometrium, leading to necrosis and endometrial dissection. Finally, blood vessels rupture and a mixture of blood and exfoliated endometrium fragments are formed. The main pathogenesis of EMS, including implant theory, menstrual blood reflux theory, endometrial determinism, and stem cell theory, all began with the shedding of endometrial fragments into the pelvic cavity. Under normal circumstances, the shedding endometrial cells immediately convert into a state of severe hypoxia when the blood supply of the endometrial capillaries is lost. Then relevant apoptotic signals are activated to induce apoptosis and endometrial cells are finally cleared by the immune cells in the pelvic. At the same time, this transient physiological hypoxia could promote the timely repair of the exfoliated endometrial surface and prevent excessive menstrual bleeding, which is mostly meditated by hypoxia-induced high expression of vascular endothelial growth factor (VEGF) (20). However, in patients with EMS, various changes of cell biological functions (21) may occur in the shedding endometrial cells under hypoxic stress, such as increased adhesive and invasive capacity, enhanced angiogenesis, and dysregulated immuno-clearance system, thus improving their ability to resist hypoxia or activating certain functions to resist apoptosis, adapt to the hypoxic environment, and survive. Eventually, they will develop into EMS lesions. Therefore, hypoxia can also be the driving factor for EMS, but the relevant pathological mechanism remains unclear.

## The role of hypoxia-inducible factors in endometriosis

**Figure 2:**
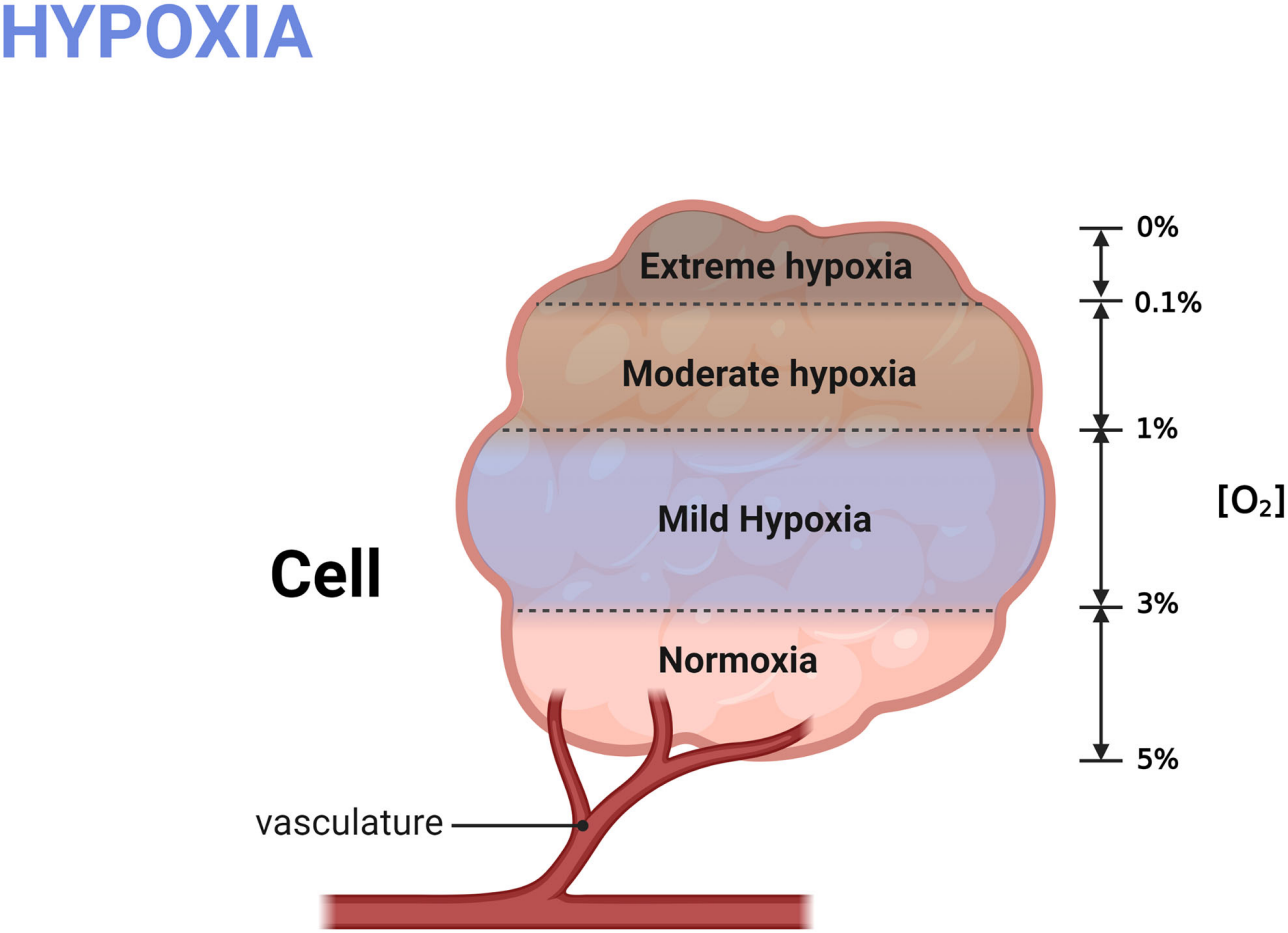
Grading of hypoxia. The normal oxygen content in human cell is 3%~5%; mild hypoxia refers to the oxygen concentration raging in 1%-3%; Oxygen content less than 1% is defined as severe hypoxia. Created with BioRender.com.

Hypoxia (22) generally refers to the pathological process in which oxygen is not adequately supplied or consumed excessively, resulting in insufficient oxygen concentration, reduced availability, and the inability to maintain normal cellular functions. If hypoxia persists, it will lead to a metabolic crisis and ultimately threaten the cell’s survival. The concentration of oxygen in the atmosphere is approximately 20%, but the content of oxygen in human tissues is much lower. Generally, the normal oxygen content of human tissues is approximately 3%~5% (as shown in **Figure 2**) (23), which is essential for the maintenance of cells’ normal life. When the intracellular oxygen content ranges in 1%~3%, it is called mild hypoxia. In this condition, molecular O2 will play a role as a critical signal to regulate cell fate, activating various adaptive mechanisms to promote cell survival and proliferation.

However, in a state of moderate hypoxia, which means that the oxygen content is less than 1%, many cells can still survive. To survive in a moderate hypoxic environment, the expression of various genes is needed to allow cells to adapt to the stress response induced by hypoxia. This process is heavily controlled by the hypoxia-inducible factors (HIFs) family. HIFs, first discovered by Semenza et al. (24) in 1992, are activated through a transcription mechanism under hypoxic conditions and then promote the expression of many hypoxia-regulated gene products to form an adaptive mechanism for cell protection. Acting as the master switch in the body in response to hypoxia, the downstream mechanisms of HIFs are multifaceted (25). For example, HIFs promotes increased secretion of erythropoietin to accelerate red blood cell production, enhancing tissue’s ability to transport oxygen, ensuring that cells have an adequate supply of oxygen (26). In addition, switching of metabolic patterns during hypoxia is essential to reduce oxygen consumption. HIFs can activate glycolytic genes and inhibit tricarboxylic acid cycle metabolism, thereby reducing oxygen consumption and maintaining cell survival in hypoxia condition (27). HIFs can also induce increased secretion of vascular endothelial growth factor (VEGF) in cells and increase the blood supply of tissues by promoting angiogenesis, thus protecting cells from ischemic damage (28). Hypoxia can also promote the synthesis of some glucose transporters and maintain the production of high-energy molecular ATP in cells (29). Overall, HIFs are the most sensitive and important nuclear transcription regulators response to hypoxia. They are prevalent in various mammalian cells even in the simplest animal (30). HIFs regulate the expression of various genes, and they are widely involved in regulating oxygen homeostasis in response to hypoxia and other changes in the cell’s internal environment of the cell. Thus, HIF-1 is a key oxygen sensor and a hypoxic adaptive response regulator.

Regarding EMS, when endometrial cells enter the pelvic cavity with retrograde menstrual blood and have not established effective blood circulation, continuous hypoxia can induce the overexpression of HIF-1 in endometrial cells, thus inducing the secretion of various factors within the endometrial environment and prompting the development of endometriosis through multiple mechanisms (31). Angiogenesis is the primary challenge for the survival of endometrial cells flowed into the pelvis, and it is also a major prerequisite for the initiation and progression of endometriosis (32). In this process, HIFs can induce the expression of numerous downstream factors to promote the angiogenesis of endometrial cells. Among them, VEGF family are the most classical factors which were found up-regulated both *in vivo* and *in vitro (* 33, 34). Other cytokines and chemokines, such as IL-8, leptin, CYR61 and osteopontin also participant in the HIF-induced angiogenesis process (35–37). In addition, HIFs could also enhance cell adhesive ability through the regulation of Transforming growth factor β1 (TGF-β1), Enhancer of zeste homolog 2 (EZH2), and anthrax toxin receptor 2 (ANTXR2) (34, 38). Hypoxia also participants in the regulation of inflammation and immune system *via* mediating the expression of IL-6, DUPS2, and COX-2 in endometrial cells (39). Furthermore, although EMS is a benign disorder, it displays some pathogenic characteristics of malignant diseases (40), such as the tendency to invade and relapse, and adaptation to hypoxia (41). A latest research showed that the expression of early growth response 1 (EGR1) and its downstream target carbonic anhydrase 9 (CA9), which were up-regulated in hypoxic cancers, are significantly increased in ectopic lesions (42). In addition, malignant cells possess a unique energy metabolism feature which is called the “Warburg effect” (43), also known as aerobic glycolysis. Specifically, even in anoxic conditions, malignant tumor cells could remain powered by glycolysis, increasing glucose consumption and lactic acid production. This progress also provides abundant energy for the growth of malignant cells, ensuring their ability to proliferate rapidly, and HIF-1α plays a vital role in this energy metabolic pathway and may protect cells from hypoxia (44). Interestingly, Young et al. (45) found that the “Warburg effect” also existed in the EMS lesion, and HIF-1α also showed same effect on the aerobic glycolysis of EMS.

## Hypoxia activates the unfolded protein response

Recent evidence suggests that HIFs alone cannot achieve the whole program of adaptive changes required for cell survival under hypoxic stress and the unfolded protein response (UPR) under endoplasmic reticulum stress (ERS) plays an essential complementary role in this process (46, 47). UPR and HIF pathway interacts with HIF-independent pathways, forming a highly correlated regulatory network under hypoxia stress.

The endoplasmic reticulum (ER) is an extensive intracellular membranous network extending to the entire cytoplasm. The key site for lipid and glucose metabolism, calcium homeostasis, detoxifying drugs, and metabolisms is the central processing unit responsible for protein translation, folding, and modification (48). In protein synthesis, folding the protein spatial structure depends on the oxygen content, which is also called oxidizing protein folding (49). When intracellular oxygen availability is reduced (hypoxia), protein folding will be disturbed, leading to accumulation of misfolded or unfolded proteins. These changes break the protein dynamics in the endoplasmic reticulum and activate the UPR signaling network, thus inducing the production of self-protection mechanisms in cells. This process is called endoplasmic reticulum stress (ERS), which aims to restore homeostasis and function of the intracellular environment (50–52).

**Figure 3:**
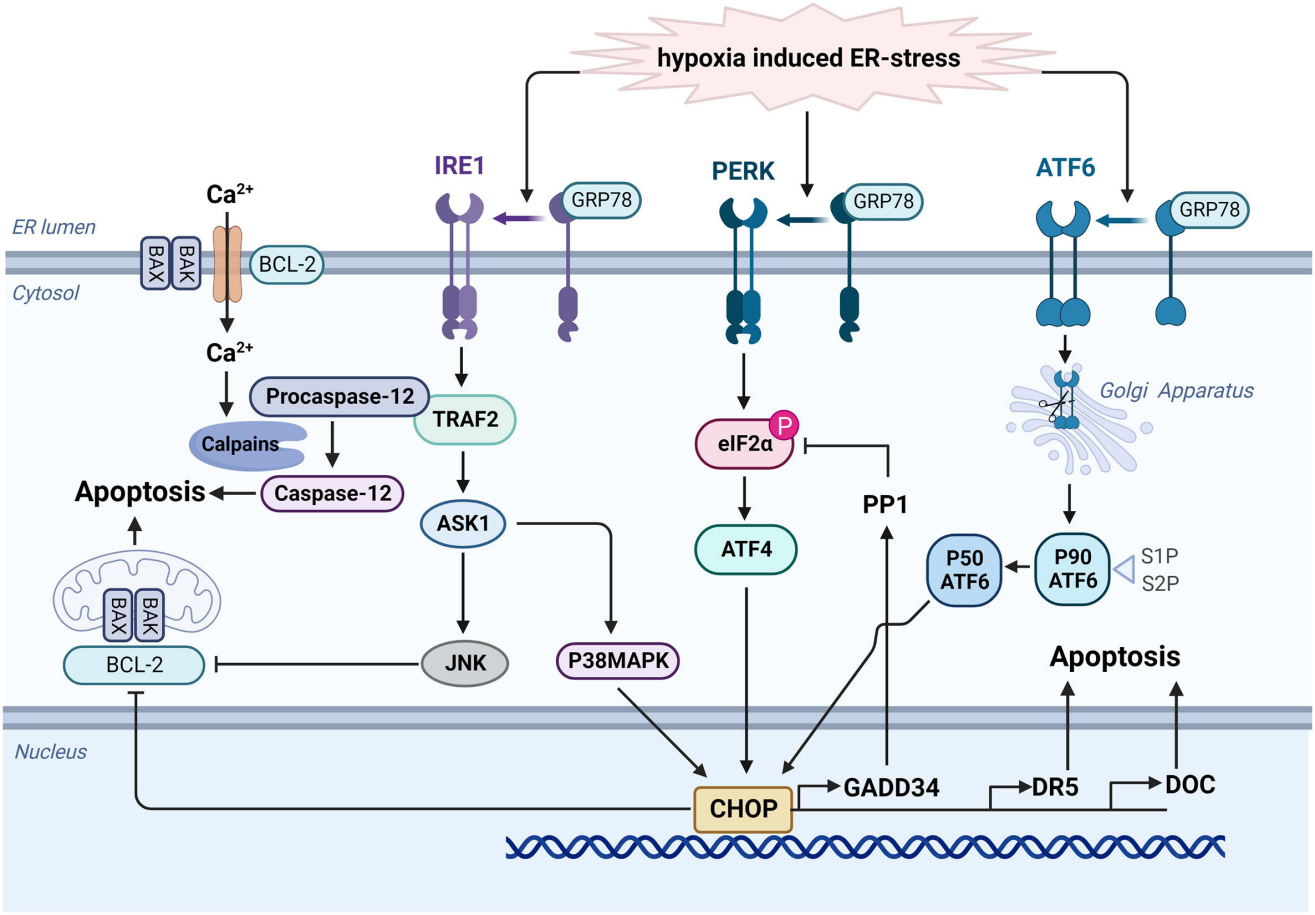
Hypoxia activates the UPR signaling network to mediate apoptosis. When ERS damage occurs, GRP78 dissociates from PERK, ATF6 and IRE1α. Activated ATF6 can be transported to Golgi apparatus. After modification, it could induce CHOP expression and lead to UPR-related apoptosis *via* the inhibition of BCL-2 protein family and the activation of multiple genes including GADD34, DR5, and DOC. Similarly, activated PERK phosphorylates the α subunit of eIF2 and initiates translation of ATF4, then promoting the transcription of CHOP. Additionally, activated IRE1α stimulates the expression of TRAF2, then activate ASK1 and JNK to promote apoptosis. TRAF2 also promotes clustering of procaspase-12, and it will be activated into caspase 12 by calpains under ER stress. Created with BioRender.com.

UPR is mediated by a partner molecule specific for the endoplasmic reticulum, namely glucose-regulated protein78(GRP78), and three transmembrane protein stress sensors (53), namely protein kinase RNA-like endoplasmic reticulum kinase (PERK), activating transcription factor 6 (ATF6) and inositol-requiring protein 1α (IRE1α)(as shown in **Figure 3**). UPR can avoid the ERS-caused damage by reducing the accumulation of unfolded proteins and improving the correct folding of proteins, and restoring normal physiological functions of cells. However, if ERS becomes persistent or damage is too severe, the signal will change from pro-survival to pro-apoptosis (54). Under normal physiological conditions, these three UPR-related transmembrane proteins all bind to GRP78 and remain in an inactive state. When ERS damage occurs, GRP78 will dissociate from these transmembrane proteins (55). Activated PERK phosphorylates the α subunit of eukaryotic initiation factor 2 (eIF2), initiating selective translation of activating transcription factor 4 (ATF4) under stress conditions. ATF4 is mainly involved in regulating peptide chain biosynthesis, antioxidant action, protein folding, and gene expression related to the maintenance of redox homeostasis (56). If the stress persists, ATF4 can also promote the transcription of C/EBP homologous protein (CHOP), a pro-apoptotic protein, to induce cell apoptosis (57). Activated ATF6 can also initiate related transcriptional procedures to restore endoplasmic reticulum homeostasis, including inducing GRP78 expression, promoting protein chaperone and lipid synthesis, stimulating endoplasmic reticulum degradation, and improving N-glycosylation (58, 59), and ATF6 also induces CHOP expression, leading to UPR-related apoptosis (60, 61).

Activated IRE1α regulates gene expression by increasing ER protein folding capacity *via* TRAF2. It also promotes the expression of proteins related to disulfide bond formation and molecular chaperones and proteins involved in ER degradation and vesicle transport (62). TRAF2 also promotes clustering of procaspase-12. Under ER stress, sustained Ca2+ release from the ER and activate calpains, which may induce the activation of caspase 12 to mediate apoptosis (63). In addition, IRE1α kinase can up-regulated the expression of apoptosis signal regulated kinase 1(ASK1) and then activate c-Jun N-terminal kinase (JNK) to promote apoptosis through the inhibition of BCL-2 protein family (64, 65). CHOP, a member of C/EBP transcription factor family, is an ERS-specific effector molecule representing an important cell transition signal to apoptosis (66). Under normal physiological conditions, CHOP expression remains at a very low level, while in the ERS state, activation of PERK, ATF6, and IRE1α can induce CHOP transcription, significantly increasing its expression and migration into the nucleus, promoting cell apoptosis *via* the inhibition of BCL-2 protein family (67, 68). CHOP also prompt the expression of GADD34 (69), which complexes with protein phosphatase 1 (PP1) to induce the dephosphorylation of eIF2α, thus forming a negative feedback loop. Other downstream target genes, including death receptor 5 (DR5) and downstream of CHOP (DOC) have also been proposed to lead to apoptosis (70).

Since protein synthesis and oxygen-dependent protein folding are energy-intensive processes, and hypoxia significantly reduces the level of intracellular ATP, regulating mRNA translation is an important cellular response to hypoxia (27). Hypoxia activates PERK, leading to the inhibition of eIF2α phosphorylation and overall translation, while ATF4 translation increases after PERK/eIF2α is activated (71, 72). This is a rapid response independent of HIF-1α and usually occurs within minutes of exposure to hypoxic conditions in cells. The phosphorylation of eIF2α is transient, attributed to the negative feedback caused by GADD34 dependent on ATF4 upregulation. Dephosphorylation of eIF2α will increase the production of various intracellular reactive oxygen species (ROS), thus stimulating various biological reactions, while mitochondria are the main source of oxygen-deficient ROS (73). Mitochondrial hypoxic ROS activates and integrates the stress response to maintain energy and REDOX homeostasis and then constitutes an early adaptive response to hypoxia. Some enzyme antioxidants, such as catalase and glutathione peroxidase, can reduce eIF2α phosphorylation due to hypoxia. In contrast, ATF4 can promote cell survival by enhancing the upregulation of HIF-1α-mediated downstream targets (74). Brief exposure to ERS can modulate cells and enable them to survive in more severe stress. Survival-promoting genes may induce this pre-adaptation, and integration of stress response is also a sufficient survival-promoting mechanism under hypoxia. Cells with impaired PERK-eIF2a-ATF4 signal transduction are more sensitive to hypoxic stress *in vitro*, indicating that the PERK-eIF2a-ATF4 pathway provides a survival advantage for cells under hypoxic conditions (75, 76), which is crucial for resistance to intracellular hypoxia, metabolic stress, and starvation.

Scientists have analyzed variations of gene expression under hypoxia conditions, but UPR-related genes can be excessively induced in the state of “extreme” hypoxia or “moderate” hypoxia. For example, X-box binding protein 1 (XBP1), a transcription factor containing zinc finger structure, is critical for UPR signaling network and acts on the folding of multiple proteins regulated by downstream target molecules. Under normal conditions, XBP1 exists in XBP1 unspliced (XBP1u). In the hypoxia state, XBP1u is activated into XBP1 spliced (XBP1s) by IRE1, enabling effective transcription of multiple target genes in the nucleus. Meanwhile, hypoxia can also induce XBP1 expression and activate its mRNA splicing in a HIF-1α dependent pathway, leading to increased XBP1s (77). When XBP1s were co-localized in tumors with hypoxia markers, the loss of XBP1 increased the sensitivity of transformed cells to hypoxia-induced apoptosis and inhibited tumor growth (78). Studies have demonstrated that XBP1 is critical for carcinogenicity and progression of triple-negative breast cancer (TNBC). TNBC is a type of breast cancer that lacks estrogen receptors, progesterone, and HER2, in which HIF-1α is overactivated. However, XBP1 splicing is not directly regulated by HIF-1α. XBP1 mainly enhances the transcriptional activity of HIF-1α and regulates its transcriptional program by binding to HIF-1α and forming the transcription complex to drive the carcinogenicity of TNBC. In contrast, XBP1 knockout can reduce the formation of breast cancer lesions under hypoxia conditions. The characteristics of XBP1 gene expression in TNBC patients are closely related to those in the state of hypoxia, indicating a poor prognosis.

## The role of UPR in EMS

UPR is a protection mechanism to maintain the stability of the intracellular environment under hypoxia circumstances, and its activation will promote cell survival. To determine the role of UPR in the pathogenesis of EMS, Guzel (79) et al. detected GRP78 expression in normal and ectopic endometrial cells, finding that the level of GRP78 in ectopic endometrial cells is significantly higher than that of normal endometrial cells. This indicated that the UPR cascade reaction was activated in EMS, and the upregulated expression of GRP78 significantly reduced the sensitivity of ectopic endometrial cells to apoptosis and increased their anti-apoptosis ability, which was beneficial for the survival of ectopic endometrial cells. Other UPR proteins, p-IRE1 and p-PERK, were also found increased in endometrial cells when they were treated with peritoneal fluid obtained from women with endometriosis (80). Taylor (81) et al. also reported that UPR induced the increased expression of IL-8, indicating that UPR may be involved in the pathogenesis of EMS by promoting neovascularization and cell survival *via* IL-8 related pathways. The protein kinase B (AKT)/mammalian target of rapamycin (mTOR) signaling pathway plays an essential role in enhancing the invasiveness of cells, and inhibition of this pathway can effectively reduce the invasiveness of various cancer cells (82). Several studies (83, 84) have displayed that UPR can inhibit AKT/mTOR pathway through CCAAT/CHOP/(Tribbles Homolog 3, TRIB3) signal transduction and regulate the invasiveness of ectopic endometrial cells. EMS is an estrogen-dependent disease and is related to progesterone resistance (85). UPR-induced apoptosis can be inhibited by estrogen, thus promoting endometrial cell survival (86). In contrast, in the secretory phase, due to the antagonistic effect of progesterone, the inhibition effect of estrogen on the UPR-mediated apoptotic pathway can be reduced. Then, UPR upregulation may help reduce the invasiveness of endometrial cells (87). However, progesterone resistance exists in ectopic and endometrial stromal cells of women with EMS, and in the secretory phase, progesterone resistance in ectopic endometrium stromal cells may alter the effect induced by UPR as described above (88, 89). *In vitro* studies on UPR regulating endometrial cell invasiveness also suggest that (90), in endometrial cells with estrogen added alone in the proliferative phase, the expressions of GRP78, CHOP, and TRIB3 increased significantly with increased progesterone during the secretory phase. In contrast, endometrial cell invasiveness was significantly suppressed when AKT and mTOR activity was inhibited. Thus, progesterone can upregulate the expression of UPR-related CHOP and TRIB3 by antagonizing estrogen and inhibiting the AKT/mTOR pathway, reducing the invasiveness of endometrial cells. These progesterone-induced signal regulations can occur in normal endometrial cells. In ectopic cells, progesterone has no significant effect on CHOP/TRIB3 or AKT/mTOR signaling, so it does not play a role in the invasiveness of endometrial cells.

## Discussion

EMS severely affects the physical and mental health and quality of life of women of reproductive age. The implantation theory on the pathogenesis of EMS indicates the key role of hypoxia in the occurrence and development of EMS, which involves cell biological processes such as apoptosis, adhesion, proliferation, invasion, and metastasis of ectopic endometrial cells. The mechanism of EMS is complex, and its etiology and pathology cannot be clearly explained with a onefold theory. Hypoxia-induced UPR may be one of the potential mechanisms by which ectopic endometrium cells could resist apoptosis and develop into endometriotic lesions, and some drugs (91, 92) targeting this mechanism may become potential effective therapeutic agents for the treatment of the disease. But the exact mechanism of hypoxia-stimulated UPR in EMS remains to be explored, and we look forward to new break-throughs in this field.

## Author contributions

YZ and YJ contributed equally to the manuscript. YZ contributed to the conception and design of the review. YZ and YJ drafted the manuscript. YJ performed the descriptive figures. YW and RW revised the manuscript and provided critical advice on the content of the manuscript. All authors read and approved the final manuscript.

## Funding

This work was supported by National Natural Science foundation of China (82101723), Department of Education of Zhejiang Province (Y202045691), and Zhejiang Province Key R&D Program (2021C03095).

## Acknowledgments

We gratefully acknowledge the assistance of Home for Researchers for language modification.

## Conflict of interest

The authors declare that the research was conducted in the absence of any commercial or financial relationships that could be construed as a potential conflict of interest.

## Publisher’s note

All claims expressed in this article are solely those of the authors and do not necessarily represent those of their affiliated organizations, or those of the publisher, the editors and the reviewers. Any product that may be evaluated in this article, or claim that may be made by its manufacturer, is not guaranteed or endorsed by the publisher.
